# Pregnancy, pregnancy loss, and the risk of cardiovascular disease in Chinese women: findings from the China Kadoorie Biobank

**DOI:** 10.1186/s12916-017-0912-7

**Published:** 2017-08-08

**Authors:** Sanne A. E. Peters, Ling Yang, Yu Guo, Yiping Chen, Zheng Bian, Xiaocao Tian, Liang Chang, Shuo Zhang, Jiaqiu Liu, Tao Wang, Junshi Chen, Liming Li, Mark Woodward, Zhengming Chen, on behalf of the China Kadoorie Biobank collaboration group

**Affiliations:** 1 The George Institute for Global Health, University of Oxford, Les Gros Clark Building, South Parks Road, Oxford, OX1 3QX UK; 2 Clinical Trials Service Unit and Epidemiological Studies Unit, University of Oxford, Old Road Campus, Oxford, OX3 7LF UK; 3 Chinese Academy of Medical Sciences, Dong Cheng District, Beijing, China; 4 NCDs Prevention and Control Department, Qingdao CDC, Qingdao, China; 5 Henan CDC, Zhengzhou, Henan China; 6 Suzhou CDC, Suzhou, Jiangsu China; 7 Pengzhou CDC, Pengzhou, Sichuan China; 8 Maiji CDC, Tianshui, Gansu China; 9 China National Center for Food Safety Risk Assessment, Chaoyang District, Beijing, China; 10 Department of Public Health, Beijing University, Beijing, China; 11 The George Institute for Global Health, University of New South Wales, Sydney, Australia; 12 Department of Epidemiology, Johns Hopkins University, Baltimore, MD USA

## Abstract

**Background:**

Pregnancy and pregnancy loss may be linked to cardiovascular disease (CVD). However, the evidence is still inconsistent, especially in East Asians, whose reproductive patterns differ importantly from those in the West. We examined the associations of pregnancy, miscarriage, induced abortion, and stillbirth with CVD incidence among Chinese women.

**Methods:**

In 2004–2008, the nationwide China Kadoorie Biobank recruited 302,669 women aged 30–79 years from ten diverse localities. During 7 years of follow-up, 43,968 incident cases of circulatory disease, 14,440 of coronary heart disease, and 19,925 of stroke (including 11,430 ischaemic and 2170 haemorrhagic strokes), were recorded among 289,573 women without prior CVD at baseline. Cox regression yielded multiple adjusted hazard ratios (HRs) for CVD risks associated with pregnancy outcomes.

**Results:**

Overall, 99% of women had been pregnant, and among them 10%, 53%, and 7% reported having a history of miscarriage, induced abortion, and stillbirth, respectively. Each additional pregnancy was associated with an adjusted HR of 1.03 (95% confidence interval, CI: 1.02; 1.04) for circulatory disease. A history of miscarriage, induced abortion, and stillbirth, respectively, were associated with adjusted HRs of 1.04 (1.01; 1.07), 1.04 (1.02; 1.07), and 1.07 (1.03; 1.11) for circulatory disease. The relationship was stronger with recurrent pregnancy loss; adjusted HRs for each additional loss being 1.04 (1.00; 1.09) for miscarriage, 1.02 (1.01; 1.04) for induced abortion, and 1.04 (1.00; 1.08) for stillbirth.

**Conclusions:**

Among Chinese women, increases in pregnancy, and a history and recurrence of miscarriage, induced abortion, and stillbirth are each associated with a higher risk of CVD.

**Electronic supplementary material:**

The online version of this article (doi:10.1186/s12916-017-0912-7) contains supplementary material, which is available to authorized users.

## Background

Pregnancy poses a substantial challenge to the cardiovascular system of the mother [1–3], with most, but not all, studies suggesting that the number of pregnancies is positively associated with maternal risk of cardiovascular disease (CVD) [4–6]. Globally, pregnancy loss is common — up to 20% of pregnancies end in a miscarriage [7], 35 induced abortions occurred annually per 1000 women aged 15–44 years worldwide in 2010–2014 [8], and an estimated 2.6 million stillbirths occurred in 2015 [9]. Several behavioural, biological, and socioeconomic factors related to pregnancy loss are also involved in the aetiology of CVD. However, despite possible shared aetiology, the long-term effects of miscarriage, induced abortion, and stillbirth on risk of CVD remain uncertain.

A history of miscarriage or recurrent miscarriage has been linked to a higher risk of coronary heart disease (CHD), but not stroke, in previous studies, yet small study sizes, retrospective design, and inadequate adjustment for confounding factors hamper definitive conclusions [10]. Evidence relating induced abortion or stillbirth to CVD is scant and, where available, primarily comes from Western populations [11–13]. Examination of cardiovascular implications of pregnancy and pregnancy loss is particularly relevant to China, where the incidence of CVD is rising and reproductive patterns have changed considerably — not only following the introduction of the one child per family policy in the late 1970s — yet remain importantly different compared to those in the West [14].

We examined the association of pregnancy and pregnancy loss with risk of circulatory disease, CHD, and stroke, including its major subtypes, in women from the China Kadoorie Biobank (CKB) [15], a contemporary prospective cohort study in ten geographically diverse regions in China.

## Methods

### Baseline survey

Detailed information about the design of, and procedures in, CKB has previously been reported [15]. Briefly, 302,669 women and 210,222 men were recruited from five urban and five rural areas of China between June 2004 and July 2008. At the study assessment clinics, trained health workers administered a laptop-based questionnaire on demographic and socioeconomic status, lifestyle factors, and personal and family medical history. This included women’s reproductive factors, with information solicited on the number of pregnancies, miscarriages, induced abortions, and stillbirths. A range of physical measurements (e.g. height, weight, waist, blood pressure) were also taken using standard methods. A blood sample was collected for long-term storage and future analyses.

### Follow-up for morbidity and mortality

Study participants were followed for cause-specific morbidity and mortality through linkage with regional disease and death registers and with the national health insurance (HI) system. Causes of death were obtained from official death certificates and were, where necessary, supplemented by reviews of medical records. Data linkage with HI agencies was carried out every 6 months to retrieve all coded hospitalised events occurring in that period for study participants. To minimise attrition, active follow-up was performed annually. The main disease endpoints, based on the tenth edition of the International Classification of Diseases (ICD-10), for the present study were incident CHD (ICD-10 I20–I25), stroke (I60–I61, I63–I64), and all circulatory diseases (I00–I99). Haemorrhagic stroke (I61) and ischaemic stroke (I63) were secondary endpoints. Participants contributed only the first outcome (whether non-fatal or fatal) experienced during follow-up.

### Statistical analyses

The present analysis involves all women without a self-reported history of CHD or stroke at baseline (*n* = 289,573). Analyses on the association between pregnancy loss and CVD outcomes were restricted to women who had ever been pregnant (*n* = 282,797).

Baseline characteristics are presented as means (standard deviation) for continuous variables and as percentages for categorical variables. Cox proportional hazards models were used to estimate hazard ratios (HRs) and 95% confidence intervals (CIs) for incident circulatory disease, CHD, and stroke by number of pregnancies, miscarriages, induced abortions, and stillbirths. The Cox proportional hazards assumption was checked using log cumulative hazard plots and appeared to be reasonable. All analyses were stratified by age at risk and area of residence and adjusted for highest level of education attained (none, primary, secondary, tertiary or above), household income (<5000 yuan, 5000–19,999 yuan, ≥20,000 yuan), smoking (current, former, never), alcohol use (weekly, occasionally, never), physical activity, systolic blood pressure (SBP), history of hypertension, body mass index (BMI), and history of diabetes at baseline. Analyses for miscarriage, induced abortion, and stillbirth were additionally adjusted for number of live births and, where appropriate, number of miscarriages, induced abortions, and stillbirths. For comparisons involving more than two groups, CIs were estimated using floating absolute risks [16]. In analyses restricted to women who had ever been pregnant, we estimated the HRs and CIs per additional pregnancy. Similarly, the HRs and CIs per additional miscarriage, induced abortion, or stillbirth were estimated among women who had experienced at least one miscarriage, induced abortion, or stillbirth. Subgroup analyses were conducted to obtain the HRs and CIs for incident circulatory disease, CHD, and stroke per additional pregnancy, miscarriage, induced abortion, or stillbirth by study region, age group, highest level of attained education, BMI, smoking status, history of diabetes, and history of hypertension. Separate models were fitted within each subgroup and tests for heterogeneity and trend were used to test for differences between subgroups. The heterogeneity test tests the null hypothesis that the coefficients of the variable of interest are zero for all subgroups against the alternative that at least one of them is not. The test statistic has a chi-squared distribution because it is a sum of independent *N*(0, 1) variables, due to its use of floating standard errors. The trend test tests whether the (independent normally distributed) coefficients follow a linear trend, for which the test statistic has a chi-squared distribution with one degree of freedom under the null. Analyses were performed using SAS version 9.3 and R version 3.1.2.

## Results

Overall, the mean baseline age was 57 years, and 99% of women had ever been pregnant (Table 1). Among those who had ever been pregnant, 10% had a history of miscarriage, 53% had a history of induced abortion, and 7% had a history of stillbirth. Although the baseline characteristics of women who had been pregnant only once were generally more favourable compared to those of women who had never been pregnant or who had been pregnant multiple times, stratification for age and study area largely attenuated these differences (Additional file 1: Table S1). Women with a history of miscarriage had a lower prevalence of induced abortion or stillbirth compared to women without a history of miscarriage (Additional file 1: Table S2). Women with a history of induced abortion had a lower prevalence of miscarriage or stillbirth compared to women without a history of induced abortion (Additional file 1: Table S3). The prevalence of miscarriage was higher and the prevalence of induced abortion was lower among women with a history of stillbirth compared to women without a history of stillbirth (Additional file 1: Table S4).

**Table 1:** Baseline characteristics of study participants by number of pregnancies

	Total	0 pregnancies	1 pregnancy	2 pregnancies	3 pregnancies	4 pregnancies	≥5 pregnancies
Number of women (*N*)	289,573	2809 (1.0)	26,891 (9.3)	76,137 (26.3)	76,802 (26.5)	50,779 (17.5)	56,155 (19.4)
Rural, %	56.8	40.1	37.9	56.3	58.4	59.6	61.0
Age, years	50.5 (10.3)	49.9 (11.5)	44.2 (7.7)	46.1 (8.0)	49.4 (9.0)	53.1 (9.9)	58.8 (10.4)
Education level, %							
Primary or below	56.8	43.3	32.6	49.3	56.1	62.6	74.7
Secondary or above	43.2	56.7	67.4	50.7	43.9	37.4	25.3
Household income, %							
Low	10.2	12.1	5.3	6.8	8.4	11.5	18.4
Middle	49.1	52.3	43.7	46.9	48.9	50.6	53.5
High	40.7	35.6	51.1	46.4	42.8	37.9	28.1
Current smoking, %	4.9	6.7	3.4	2.9	4.0	5.6	8.9
Regular alcohol use, %	36.5	42.1	41.7	35.7	36.3	36.4	35.2
Physical activity (MET hours/day)	17.2 (11.0, 28.7)	14.8 (9.3, 24.7)	20.3 (12.8, 31.8)	20.4 (12.3, 32.3)	18.3 (11.2, 30.0)	15.7 (10.3, 26.6)	12.9 (8.4, 21.1)
Systolic blood pressure, mmHg	129.4 (21.8)	127.7 (23.0)	123.0 (18.6)	126.1 (19.9)	128.7 (21.3)	131.6 (22.3)	136.0 (23.8)
Body mass index, kg/m^2^	23.8 (3.4)	23.4 (3.8)	23.6 (3.3)	23.7 (3.3)	23.8 (3.4)	23.9 (3.5)	23.8 (3.6)
History of hypertension, %	10.2	9.0	5.5	7.2	9.7	12.4	15.6
History of diabetes, %	2.9	2.7	1.4	1.8	2.6	3.3	4.9
Pregnancy loss, %							
History of miscarriage	9.8	–	0.6	2.3	7.3	12.2	21.2
History of induced abortion	52.8	–	1.3	44.4	60.2	65.1	65.1
History of stillbirth	6.5	–	0.3	1.2	4.0	7.2	14.7
Ever use of oral contraceptives, %	9.8	2.2	5.6	8.4	10.9	11.7	11.0

Values are percentages for categorical variables, and means and standard deviations for continuous variables, expect for physical activity where median and 25th and 75th percentiles are shown

*MET* metabolic equivalent

During a median of 7.1 years (Q1: 6.2; Q3: 8.1) follow-up, 43,968 incident cases of circulatory disease were recorded, including 14,440 cases of CHD, 19,925 cases of stroke, 11,430 cases of ischaemic stroke, and 2170 cases of haemorrhagic stroke.

### Pregnancy and CVD risks

**Fig. 1:**
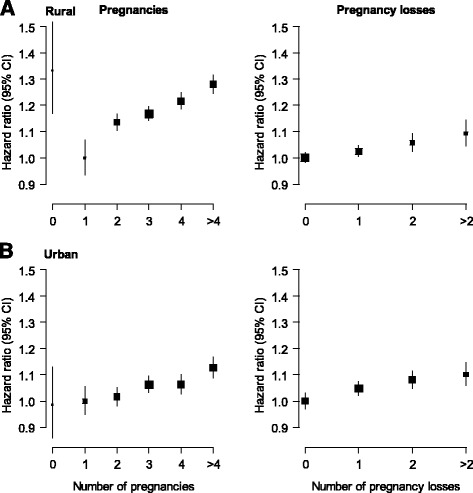
Adjusted hazard ratios (95% confidence intervals (CIs)) for incident circulatory disease associated with number of pregnancies and pregnancy losses. Analyses are stratified by age at risk and study area and adjusted for level of attained education, household income, smoking status, alcohol use, systolic blood pressure, history of hypertension, physical activity, body mass index, and history of diabetes. Analyses for pregnancy loss are additionally adjusted for number of live births. The hazard ratios are plotted on a floating absolute scale. Each square has an area inversely proportional to the standard error of the log risk. Vertical lines indicate the corresponding 95% CIs. Analyses for pregnancy loss are among women with at least one pregnancy

**Fig. 2:**
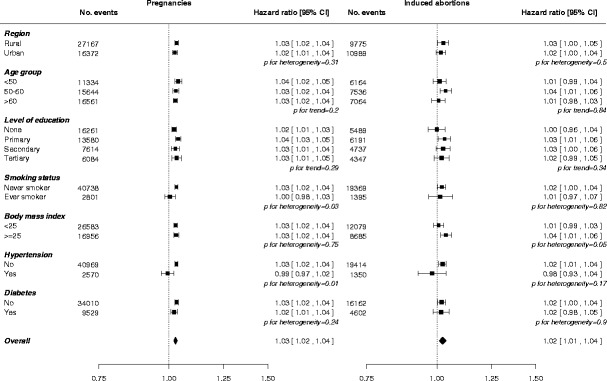
Adjusted hazard ratios for incident circulatory disease per additional pregnancy (left) and induced abortion (right) by baseline characteristics. Adjustments are as in Table 2. Each square has an area inversely proportional to the standard error of the log risk. The diamond indicates the overall risk of circulatory disease per additional pregnancy and induced abortion and its 95% CI. Analyses for pregnancy and induced abortion, respectively, are among women with at least one pregnancy or at least one induced abortion

There was no difference in the risk of circulatory disease comparing gravid women to nulligravid women; adjusted HR (95% CI) 0.98 (0.89; 1.07). However, in gravid women, there was a log-linear association between the number of pregnancies and the risk of circulatory disease (Table 2 and Additional file 1: Figure S1), with adjusted HRs of 1.00 (0.96; 1.04), 1.07 (1.04; 1.09), 1.10 (1.08; 1.12), 1.13 (1.11; 1.15), and 1.19 (1.16; 1.22) for one (reference group), two, three, four, and five or more pregnancies, respectively. This dose-response relationship was consistent between women from rural and urban areas (Fig. 1). Each additional pregnancy was associated with a 1.03 (1.02; 1.04) higher risk of circulatory disease, with some indication of stronger effects among ever smokers or those with a history of hypertension (Fig. 2). Findings were similar in the analyses for CHD, stroke, and stroke subtypes (Table 2, Additional file 1: Table S5, and Figures S2 and S3).

**Table 2:** Adjusted hazard ratios (95% confidence intervals) for incident coronary heart disease, stroke, and circulatory disease associated with number of pregnancies, miscarriages, induced abortions, and stillbirths

	CHD		Stroke		Circulatory disease	
	No. events	HR (95% CI)	No. events	HR (95% CI)	No. events	HR (95% CI)
Pregnancies						
*Ever vs. never*		*0.91 (0.78; 1.06)*		*0.98 (0.86; 1.12)*		*0.98 (0.89; 1.07)*
None	166	1.20 (1.03; 1.40)	211	1.06 (0.93; 1.22)	429	1.14 (1.04; 1.26)
1	779	1.00 (0.93; 1.08)	994	1.00 (0.94; 1.07)	2333	1.00 (0.96; 1.04)
2	2534	1.01 (0.96; 1.05)	3501	0.99 (0.95; 1.02)	8915	1.07 (1.04; 1.09)
3	3306	1.03 (1.00; 1.07)	4718	1.02 (0.99; 1.05)	11,193	1.10 (1.08; 1.12)
4	3021	1.14 (1.10; 1.18)	4064	1.03 (1.00; 1.06)	8804	1.13 (1.11; 1.15)
≥ 5	4634	1.19 (1.15; 1.24)	6437	1.09 (1.05; 1.13)	12,294	1.19 (1.16; 1.22)
*Per additional* ^*a*^		*1.04 (1.03; 1.05)*		*1.02 (1.02; 1.03)*		*1.03 (1.02; 1.04)*
Miscarriages						
*Ever vs. never* ^*b*^		*1.07 (1.02; 1.13)*		*1.04 (1.00; 1.09)*		*1.04 (1.01; 1.07)*
None	12,629	1.00 (0.98; 1.02)	17,497	1.00 (0.98; 1.02)	39,089	1.00 (0.99; 1.01)
1	1220	1.04 (0.98; 1.10)	1695	1.04 (0.99; 1.09)	3390	1.01 (0.98; 1.05)
2	304	1.19 (1.06; 1.33)	374	1.09 (0.99; 1.21)	779	1.12 (1.05; 1.21)
≥ 3	121	1.26 (1.05; 1.51)	148	1.15 (0.98; 1.36)	281	1.12 (0.99; 1.26)
*Per additional* ^*a*^		*1.07 (1.01; 1.14)*		*1.04 (0.98; 1.10)*		*1.04 (1.00; 1.09)*
Induced abortions						
*Ever vs. never* ^*b*^		*1.11 (1.06; 1.15)*		*1.04 (1.01; 1.07)*		*1.04 (1.02; 1.07)*
None	7334	1.00 (0.97; 1.03)	10,071	1.00 (0.98; 1.02)	22,775	1.00 (0.98; 1.02)
1	3714	1.08 (1.05; 1.11)	5217	1.04 (1.01; 1.06)	11,834	1.04 (1.02; 1.06)
2	2160	1.13 (1.08; 1.18)	2904	1.05 (1.01; 1.09)	6032	1.05 (1.02; 1.07)
≥ 3	1066	1.14 (1.07; 1.21)	1522	1.09 (1.04; 1.15)	2898	1.09 (1.05; 1.13)
*Per additional* ^*a*^		*1.02 (1.00; 1.05)*		*1.03 (1.00; 1.05)*		*1.02 (1.01; 1.04)*
Stillbirths						
*Ever vs. never* ^*b*^		*1.00 (0.94; 1.07)*		*1.06 (1.01; 1.12)*		*1.07 (1.03; 1.11)*
None	13,078	1.00 (0.96; 1.04)	17,846	1.00 (0.97; 1.03)	40,052	1.00 (0.98; 1.02)
1	822	0.96 (0.90; 1.02)	1355	1.05 (1.00; 1.11)	2538	1.05 (1.01; 1.09)
2	240	1.08 (0.95; 1.23)	368	1.13 (1.02; 1.25)	662	1.12 (1.04; 1.21)
≥ 3	134	1.31 (1.10; 1.56)	145	0.99 (0.84; 1.17)	287	1.13 (1.01; 1.28)
*Per additional* ^*a*^		*1.13 (1.06; 1.20)*		*1.02 (0.96; 1.08)*		*1.04 (1.00; 1.08)*

HRs are stratified by age and study area, and adjusted for level of attained education, household income, smoking status, alcohol use, systolic blood pressure, history of hypertension, physical activity, body mass index, and history of diabetes. Analyses for miscarriage, induced abortion, and stillbirth were additionally adjusted for number of live births, and where appropriate, number of miscarriages, induced abortions, and stillbirths. ^a^Analyses are restricted to women with at least one pregnancy, miscarriage, induced abortion, or stillbirth, respectively. ^b^Analyses are restricted to women with at least one pregnancy

### Miscarriage and CVD risks

**Fig. 3:**
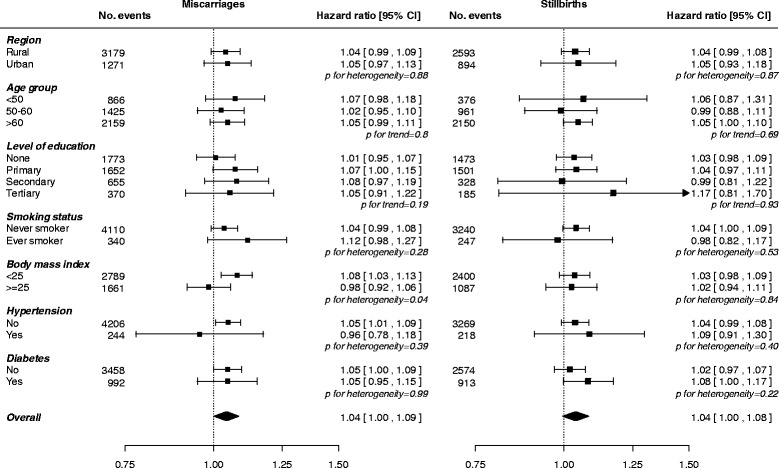
Adjusted hazard ratios for incident circulatory disease per additional miscarriage (left) and stillbirth (right) by baseline characteristics. Adjustments are as in Table 2. Each square has an area inversely proportional to the standard error of the log risk. The diamond indicates the overall risk of circulatory disease per additional miscarriage and stillbirth and its 95% CI. Analyses for miscarriage and stillbirth, respectively, are among women with at least one miscarriage or at least one stillbirth

Compared to women who had never had a miscarriage, women who had a history of miscarriage had an adjusted HR of 1.04 (1.01; 1.07) for circulatory disease. The adjusted HRs of circulatory disease associated with number of miscarriages were 1.01 (0.98; 1.05) for one, 1.12 (1.05; 1.21) for two, and 1.12 (0.99; 1.26) for three or more miscarriages (Table 2 and Additional file 1: Figure S1). The HR of circulatory disease for each additional miscarriage was 1.04 (1.00; 1.09), with little evidence of differences between subgroups of populations (Fig. 3). The strength of the association between miscarriage and risk of CHD and stroke was similar in direction, yet stronger in magnitude for CHD compared to that for stroke and total circulatory disease (Table 2 and Additional file 1: Figure S2). The HRs for CHD and stroke per additional miscarriage were 1.07 (1.01; 1.14) and 1.04 (0.98; 1.10), respectively, and were broadly consistent across different subgroups (Additional file 1: Figure S4). Analyses by stroke subtype showed that the association was predominantly driven by a higher risk of haemorrhagic, but not of ischaemic, stroke (Additional file 1: Table S5).

### Induced abortion and CVD risks

Compared to women who had never had an induced abortion, women with a history of induced abortion had an adjusted HR of 1.04 (1.02; 1.07) for circulatory disease. There was a log-linear association between the number of induced abortions and the risk of circulatory disease; the HRs were 1.04 (1.02; 1.06) for one, 1.05 (1.02; 1.07) for two, and 1.09 (1.05; 1.13) for three or more induced abortions (Table 2 and Additional file 1: Figure S1). Corresponding HRs for CHD were 1.08 (1.05; 1.11), 1.13 (1.08; 1.18), and 1.14 (1.07; 1.21), while for stroke they were 1.04 (1.01; 1.06), 1.05 (1.01; 1.09), and 1.09 (1.04; 1.15). Adjusted HRs for each additional induced abortion were 1.02 (1.01; 1.04) for circulatory disease, 1.02 (1.00; 1.05) for CHD, and 1.03 (1.00; 1.05) for stroke, with little heterogeneity across different population subgroups (Fig. 3 and Additional file 1: Figure S5). Analyses by stroke subtype yielded similar findings for ischaemic stroke, but no associations were found for haemorrhagic stroke (Additional file 1: Table S5).

### Stillbirth and CVD risks

A history of stillbirth was associated with a higher risk of circulatory disease (1.07, 1.03; 1.11) and stroke (1.06, 1.00; 1.12), yet there was no apparent association with CHD (1.00, 0.94; 1.07) (Table 2). The HR for circulatory disease associated with the number of stillbirths was 1.05 (1.01; 1.09) for one, 1.12 (1.04; 1.21) for two, and 1.13 (1.01; 1.28) for three or more stillbirths (Table 2 and Additional file 1: Figure S1). The corresponding HRs for CHD were 0.96 (0.90; 1.02), 1.07 (0.94; 1.22), and 1.29 (1.08; 1.54) (Table 2 and Additional file 1: Figure S2). There was no clear dose-response association between the number of stillbirths and stroke (Table 2 and Fig. 1). The HRs associated with each additional stillbirth were 1.04 (1.00; 1.08) for circulatory disease, 1.13 (1.06; 1.20) for CHD, and 1.02 (0.96; 1.08) for stroke. Analyses by stroke subtype yielded broadly similar patterns for ischaemic stroke but somewhat stronger associations for haemorrhagic stroke (Additional file 1: Table S5). There were no material differences across population subgroups in the association between each additional stillbirth and the risk of circulatory disease, CHD, or stroke (Fig. 3 and Additional file 1: Figure S6).

Analyses stratified by age at risk and study area only yielded similar results compared to those from the main analyses for all comparisons (Additional file 1: Tables S5 and S6).

## Discussion

This large study of almost 300,000 women in China provides a comprehensive assessment of the relationships of pregnancy, pregnancy loss, and CVD incidence in later life. There was a J-shaped relationship between the number of pregnancies and CVD, with women who had never been pregnant or with multiple pregnancies being at a higher risk of CVD, compared with those who had been pregnant once. Furthermore, a history of miscarriage, induced abortion, and stillbirth each were associated with a higher risk of CVD. Although the strength of the relationships varied between types of pregnancy loss, the relationships generally became stronger with recurrent pregnancy loss. These results were not accounted for by adjustment for a range of potential confounders, and they were broadly consistent across major demographic and clinical subpopulations.

Previous studies of mostly Western populations have provided inconclusive evidence on the association between pregnancy loss and CVD risk, which may in part be limited by retrospective design, small study sizes, different exposure definitions, and varied levels of adjustment [11–13, 17–19]. A meta-analysis of ten cohort and case-control studies reported that women with a history of miscarriage are at a 45% higher risk of CHD, compared to women who have not experienced miscarriage; recurrent miscarriage was associated with a twofold risk of CHD [10]. No association was found between a history of miscarriage and future stroke. A large-scale population-based study among more than one million women in Denmark found that a history of miscarriage was associated with an about 15% higher risk of myocardial infarction and stroke, and the associations were stronger in women with repeated miscarriages [13]. Moreover, a study among 60,105 women in Scotland reported that miscarriage, but only when consecutive, was associated with a higher risk of CHD but not of stroke; HRs for CHD were 1.75 for two and 3.18 for three or more consecutive miscarriages, respectively [20].

This study concurs with previous findings and shows reliably that the risk of circulatory disease, CHD, and stroke is higher with increasing number of miscarriages. Prospective evidence on the relevance of stillbirth for the long-term risk of CVD outcomes is scarce. However, studies generally direct towards a positive relationship [12, 13, 19, 20], particularly for CHD, with adjusted HRs for CHD ranging from 1.25 among 78,000 women in the Women's Health Initiative [19] to 3.5 among 11,500 women from the EPIC-Heidelberg cohort [12]. The only study so far in a Chinese population found no evidence of a relationship between the number of miscarriages or stillbirths and the risk of coronary or stroke death, although the small number of events limited reliable assessments of these relationships [11]. The present study included a much larger number of well-characterised incident CVD events than any of these previous studies, and hence considerably expands previous findings by providing a detailed picture of the relevance of miscarriage and stillbirth on the risk of CVD in a contemporary population of Chinese women.

Miscarriage and stillbirth may be aetiologically linked to CVD through an underlying vascular pathology, particularly endothelial dysfunction, that could contribute to poor placental function during pregnancy, resulting in pregnancy loss and a higher risk of CVD [21, 22]. Indeed, a previous small-scale study reported that women with a history of recurrent pregnancy loss had more severe endothelial dysfunction compared to women who experienced uncomplicated pregnancies [22]. Autoimmune disorders, including the antiphospholipid syndrome and systemic lupus erythematosus [23, 24], are commonly implicated in the occurrence of miscarriage and stillbirth. Hence, it may be that systemic inflammatory processes associated with autoimmune conditions and the progression of atherosclerosis lead to endothelial dysfunction and, in turn, increase the risk of both pregnancy loss and CVD [25]. A genetic predisposition might be involved, as parents of women who experienced recurrent miscarriage are more likely to experience CHD compared to parents of women without such a history [26]. Factor V Leiden or prothrombin gene mutations considerably increase the risk of abnormal placentation and recurrent miscarriage, and may predispose carriers to thrombotic disease in later life [27, 28].

Research on the health sequelae of induced abortion has primarily focussed on the risks of adverse obstetric or perinatal outcomes in subsequent pregnancies. For example, induced abortion might increase the risk of low birth weight and preterm birth [29], which has been linked to maternal CVD risk in later life in several studies [30, 31], even after accounting for socioeconomic factors, smoking, and pregnancy-related complications [32]. Conversely, a history of induced abortion has been associated with a lower risk of preeclampsia in subsequent pregnancies [33, 34], which is in accordance with the protective effects of a previous birth on preeclampsia risk in later pregnancies [35, 36], but at odds with the higher risk of cardiometabolic outcomes seen among women with a history of preeclampsia [37]. We report a graded relationship between the number of induced abortions and risk of CVD outcomes, which is in contrast with findings from previous studies that found no evidence for such a relationship [11, 12].

The present study has a number of strengths, including a large sample size, prospective design, and ability to adjust for a range of potential confounders. The generalisability of our findings was enhanced by the inclusion of women from ten diverse areas in China. While our findings were robust and consistent in a comprehensive series of analyses, the effect sizes of some estimates were small and may be subject to unmeasured or residual confounding, particularly those related to physiological, cultural, or socioeconomic factors underlying the number of pregnancies and pregnancy losses. Future Mendelian randomisation studies might help to assess the causality of our findings. Furthermore, pregnancies and pregnancy losses were self-reported and, for some women, solicited several years after their reproductive age. For example, miscarriages early in pregnancy are often silent and may have been underreported. This could have led to measurement error, which, if random, would have underestimated the true strengths of the observed associations. Data on the sequence and spacing of pregnancies and pregnancy losses across the reproductive life span were not available. Nevertheless, analyses adjusted for the total number of pregnancies and pregnancy losses throughout the reproductive lifespan or restricted to women who had reported exclusively on one type of pregnancy loss yielded virtually identical results, as in previous studies [20]. Finally, our study did not collect data on CVD risk factors either before or during pregnancy that might determine pregnancy loss as well as future CVD risk. For example, women with pre-existing or pregnancy-induced conditions such as type 1 diabetes, gestational diabetes, gestational obesity, preeclampsia, or polycystic ovary syndrome might be more likely to experience pregnancy loss than women without these risk factors.

## Conclusions

In conclusion, increases in pregnancy, and a history and recurrence of miscarriage, induced abortion, and stillbirth are each associated with a higher risk of CVD outcomes in Chinese women. Further studies are needed to elucidate the physiological, behavioural, and socioeconomic factors involved. If causal, more frequent screening and timely intervention might help to delay or prevent the onset of CVD among women with large numbers of pregnancies or recurrent pregnancy loss.

## Additional file

Additional file 1: Figure S1. Adjusted hazard ratios (95% confidence intervals) for incident circulatory disease associated with number of pregnancies, miscarriages, induced abortions, and stillbirths. **Figure S2.** Adjusted hazard ratios (95% confidence intervals) for incident CHD and stroke associated with number of pregnancies, miscarriages, induced abortions, and stillbirths. **Figure S3.** Adjusted hazard ratios for incident CHD and stroke per additional pregnancy by baseline characteristics. **Figure S4.** Adjusted hazard ratios for incident CHD and stroke per additional miscarriage by baseline characteristics. **Figure S5.** Adjusted hazard ratios for incident CHD and stroke per additional induced abortion by baseline characteristics. **Figure S6.** Adjusted hazard ratios for incident CHD and stroke per additional stillbirth by baseline characteristics. **Table S1.** Adjusted baseline characteristics of study participants by number of pregnancies. **Table S2.** Baseline characteristics of study participants by number of miscarriages. **Table S3.** Baseline characteristics of study participants by number of induced abortions. **Table S4.** Baseline characteristics of study participants by number of stillbirths. **Table S5.** Adjusted hazard ratios (95% confidence intervals) for incident ischaemic and haemorrhagic stroke subtype associated with number of pregnancies, miscarriages, induced abortions, and stillbirths. **Table S6.** Adjusted hazard ratios (95% confidence intervals) for incident coronary heart disease, stroke, and circulatory disease associated with number of pregnancies, miscarriages, induced abortions, and stillbirths. (DOCX 143 kb)
