# Supplementary material for: Pregnancy, pregnancy loss, and the risk of cardiovascular disease in Chinese women: findings from the China Kadoorie Biobank
Source: BMC Med. 2017 Aug 8;15:148. doi: 10.1186/s12916-017-0912-7 (PMC5547470; doi:10.1186/s12916-017-0912-7)
Supplement: Additional file 1: Figure S1. — Adjusted hazard ratios (95% confidence intervals) for incident circulatory disease associated with number of pregnancies, miscarriages, induced abortions, and stillbirths. Figure S2. Adjusted hazard ratios (95% confidence intervals) for incident CHD and stroke associated with number of pregnancies, miscarriages, induced abortions, and stillbirths. Figure S3. Adjusted hazard ratios for incident CHD and stroke per additional pregnancy by baseline characteristics. Figure S4. Adjusted hazard ratios for incident CHD and stroke per additional miscarriage by baseline characteristics. Figure S5. Adjusted hazard ratios for incident CHD and stroke per additional induced abortion by baseline characteristics. Figure S6. Adjusted hazard ratios for incident CHD and stroke per additional stillbirth by baseline characteristics. Table S1. Adjusted baseline characteristics of study participants by number of pregnancies. Table S2. Baseline characteristics of study participants by number of miscarriages. Table S3. Baseline characteristics of study participants by number of induced abortions. Table S4. Baseline characteristics of study participants by number of stillbirths. Table S5. Adjusted hazard ratios (95% confidence intervals) for incident ischaemic and haemorrhagic stroke subtype associated with number of pregnancies, miscarriages, induced abortions, and stillbirths. Table S6. Adjusted hazard ratios (95% confidence intervals) for incident coronary heart disease, stroke, and circulatory disease associated with number of pregnancies, miscarriages, induced abortions, and stillbirths. (DOCX 143 kb) [file 12916_2017_912_MOESM1_ESM.docx]

**Additional file 1**

**Pregnancy, pregnancy loss and the risk of cardiovascular disease in Chinese women: findings from the China Kadoorie Biobank**

**eFigure legend**

**Figure S1: Adjusted hazard ratios (95% confidence intervals) for incident circulatory disease associated with number of pregnancies, miscarriages, induced abortions, and stillbirths**

Analyses are stratified by age at risk and study area, and adjusted for level of attained education, household income, smoking status, alcohol use, systolic blood pressure, history of hypertension, physical activity, body mass index, and history of diabetes. Analyses for miscarriage, induced abortion, and stillbirth are additionally adjusted for number of live births, and where appropriate, number of miscarriages, induced abortions, and stillbirths. The hazard ratios (HRs) are plotted on a floating absolute scale. Each square has an area inversely proportional to the standard error of the log risk. Vertical lines indicate the corresponding 95% confidence intervals (CIs). Analyses for miscarriage, induced abortion, and stillbirth are among women with at least one pregnancy.

**Figure S2: Adjusted hazard ratios (95% confidence intervals) for incident CHD and stroke associated with number of pregnancies, miscarriages, induced abortions, and stillbirths**

Adjustments as in Table 2. The hazard ratios (HRs) are plotted on a floating absolute scale. Each square has an area inversely proportional to the standard error of the log risk. Vertical lines indicate the corresponding 95% confidence intervals (CIs). Analyses for miscarriage, induced abortion, and stillbirth are among women with at least one pregnancy.

**Figure S3: Adjusted hazard ratios for incident CHD and stroke per additional pregnancy by baseline characteristics**

Adjustments as in Table 2. Each closed square (solid for CHD and blank for stroke) represents the risk of CHD or stroke per additional pregnancy, with its area inversely proportional to the standard error of the log risk. The diamond indicates the overall risk of CHD or stroke per additional pregnancy and its 95% CI. Analyses are among women with at least one pregnancy.

**Figure S4: Adjusted hazard ratios for incident CHD and stroke per additional miscarriage by baseline characteristics**

Adjustments as in Table 2. Each closed square (solid for CHD and blank for stroke) represents the risk of CHD or stroke per additional miscarriage, with its area inversely proportional to the standard error of the log risk. The diamond indicates the overall risk of CHD or stroke per additional miscarriage and its 95% CI. Analyses are among women with at least one miscarriage.

**Figure S5: Adjusted hazard ratios for incident CHD and stroke per additional induced abortion by baseline characteristics**

Adjustments as in Table 2. Each closed square (solid for CHD and blank for stroke) represents the risk of CHD or stroke per additional induced abortion, with its area inversely proportional to the standard error of the log risk. The diamond indicates the overall risk of CHD or stroke per additional induced abortion and its 95% CI. Analyses are among women with at least one induced abortion.

**Figure S6: Adjusted hazard ratios for incident CHD and stroke per additional stillbirth by baseline characteristics**

Adjustments as in Table 2. Each closed square (solid for CHD and blank for stroke) represents the risk of CHD or stroke per additional stillbirth, with its area inversely proportional to the standard error of the log risk. The diamond indicates the overall risk of CHD or stroke per additional stillbirth and its 95% CI. Analyses are among women with at least one stillbirth.

**Table S1: Adjusted baseline characteristics of study participants by number of pregnancies**

|  | **0 pregnancies** | **1 pregnancy** | **2 pregnancies** | **3 pregnancies** | **4 pregnancies** | **≥5 pregnancies** |
| --- | --- | --- | --- | --- | --- | --- |
| Education level, % |  |  |  |  |  |  |
| Primary or below | 51.4 | 50.1 | 55.5 | 56.6 | 56.8 | 59.1 |
| Secondary or above | 48.6 | 49.9 | 44.5 | 43.4 | 43.2 | 40.9 |
| Household income, % |  |  |  |  |  |  |
| Low | 13.7 | 9.1 | 7.9 | 8.8 | 10.0 | 11.3 |
| Middle | 55.5 | 46.6 | 45.7 | 48.2 | 50.9 | 56.1 |
| High | 30.9 | 44.3 | 46.4 | 42.9 | 39.1 | 32.5 |
| Ever smoking, % | 6.5 | 5.9 | 3.9 | 4.5 | 5.1 | 6.5 |
| Regular alcohol use, % | 39.9 | 36.9 | 33.8 | 35.9 | 37.7 | 39.8 |
| Physical activity (MET hours/day) | 19.0 | 21.3 | 21.5 | 21.2 | 20.2 | 19.0 |
| Systolic blood pressure, mmHg | 129.8 | 128.6 | 129.5 | 129.3 | 129.2 | 128.9 |
| Body mass index, kg/m^2^ | 23.6 | 23.6 | 23.7 | 23.7 | 23.8 | 23.9 |
| History of hypertension, % | 9.2 | 8.9 | 9.8 | 10.3 | 10.6 | 10.7 |
| History of diabetes, % | 2.8 | 2.1 | 2.7 | 2.8 | 2.7 | 3.4 |
| Pregnancy loss, % |  |  |  |  |  |  |
| History of miscarriage | - | 1.5 | 2.4 | 7.1 | 12.4 | 20.4 |
| History of induced abortion | - | 1.6 | 36.6 | 58.2 | 69.7 | 78.1 |
| History of stillbirth | - | 1.4 | 1.6 | 4.0 | 6.7 | 11.0 |

Data are adjusted for age and study region. Values are percentages for categorical variables and means for continuous variables. MET, metabolic equivalent

**Table S2: Baseline characteristics of study participants by number of miscarriages**

|  | **0 miscarriages** | **1 miscarriage** | **2 miscarriages** | **≥3 miscarriages** |
| --- | --- | --- | --- | --- |
| N | 261104 (90.2) | 20279 (7.0) | 3978 (1.4) | 1401 (0.5) |
| Rural, % | 55.2 | 69.7 | 74.0 | 73.6 |
| Age, years | 50.3 (10.2) | 52.8 (10.9) | 53.9 (11.5) | 55.1 (11.7) |
| Education level, % |  |  |  |  |
| Primary or below | 55.8 | 67.7 | 70.4 | 70.4 |
| Secondary or above | 44.2 | 32.3 | 29.6 | 29.6 |
| Household income, % |  |  |  |  |
| Low | 9.8 | 13.4 | 15.7 | 18.8 |
| Middle | 48.5 | 54.5 | 55.7 | 58.0 |
| High | 41.7 | 32.1 | 28.6 | 23.2 |
| Current smoking, % | 4.8 | 5.8 | 6.0 | 7.2 |
| Regular alcohol use, % | 36.6 | 35.1 | 35.5 | 35.3 |
| Physical activity (MET hours/day) | 17.4 (11.1, 28.9) | 16.0 (10.5, 27.5) | 15.1 (10.0, 26.2) | 14.4 (9.3, 26.6) |
| Systolic blood pressure, mmHg | 129.2 (21.7) | 131.7 (22.3) | 132.8 (23.0) | 134.3 (24.5) |
| Body mass index, kg/m^2^ | 23.8 (3.4) | 23.6 (3.5) | 23.6 (3.6) | 23.5 (3.6) |
| History of hypertension, % | 10.1 | 11.4 | 11.2 | 11.8 |
| History of diabetes, % | 2.8 | 3.2 | 3.2 | 3.4 |
| Pregnancies, n | 3.2 (1.6) | 4.4 (1.7) | 5.5 (2.0) | 7.1 (2.4) |
| Pregnancy loss, % |  |  |  |  |
| History of induced abortion | 54.0 | 37.4 | 28.1 | 23.6 |
| History of stillbirth | 5.2 | 8.9 | 11.0 | 13.9 |
| Ever use of oral contraceptives, % | 10.1 | 7.9 | 7.4 | 6.9 |

Values are percentages for categorical variables, and means and standard deviations for continuous variables, expect for physical activity where median and 25^th^ and 75^th^ percentile are shown. MET, metabolic equivalent

|  | **0 induced abortions** | **1 induced abortion** | **2 induced abortions** | **≥3 induced abortions** |
| --- | --- | --- | --- | --- |
| N (%) | 136737 (47.2) | 79455 (27.4) | 45862 (15.8) | 24708 (8.5) |
| Rural, % | 70.8 | 45.8 | 40.0 | 43.8 |
| Age, years | 51.6 (10.8) | 49.9 (10.0) | 49.2 (9.6) | 49.0 (9.1) |
| Education level, % | 55.8 | 68.8 | 76.7 | 83.2 |
| Primary or below | 68.5 | 49.5 | 42.4 | 43.2 |
| Secondary or above | 31.5 | 50.5 | 57.6 | 56.8 |
| Household income, % |  |  |  |  |
| Low | 14.3 | 6.4 | 5.8 | 7.1 |
| Middle | 52.5 | 44.0 | 46.1 | 51.5 |
| High | 33.1 | 49.5 | 48.1 | 41.4 |
| Ever smoking, % | 4.5 | 4.6 | 5.1 | 7.6 |
| Regular alcohol use, % | 30.8 | 38.5 | 43.7 | 47.4 |
| Physical activity (MET hours/day) | 17.8 (11.1, 29.8) | 17.2 (11.2, 28.5) | 16.7 (10.7, 27.3) | 16.6 (10.7, 26.0) |
| Systolic blood pressure, mmHg | 132.0 (22.7) | 128.1 (21.0) | 126.3 (20.4) | 125.4 (20.0) |
| Body mass index, kg/m^2^ | 23.6 (3.5) | 23.9 (3.4) | 24.0 (3.4) | 24.0 (3.4) |
| History of hypertension, % | 10.3 | 10.4 | 10.2 | 9.6 |
| History of diabetes, % | 2.6 | 3.0 | 3.1 | 3.3 |
| Pregnancies, n | 2.9 (1.7) | 3.1 (1.3) | 3.9 (1.2) | 5.5 (1.6) |
| Pregnancy loss, % |  |  |  |  |
| History of miscarriage | 12.2 | 7.1 | 4.8 | 4.7 |
| History of stillbirth | 7.9 | 3.9 | 2.9 | 2.9 |
| Ever use of oral contraceptives, % | 4.6 | 12.0 | 16.2 | 20.5 |

**Table S3: Baseline characteristics of study participants by number of induced abortions**

Values are percentages for categorical variables, and means and standard deviations for continuous variables, expect for physical activity where median and 25^th^ and 75^th^ percentile are shown. MET, metabolic equivalent

|  | **0 stillbirths** | **1 stillbirth** | **2 stillbirths** | **≥3 stillbirths** |
| --- | --- | --- | --- | --- |
| N (%) | 270820 (93.5) | 12364 (4.3) | 2565 (0.9) | 1012 (0.3) |
| Rural, % | 55.8 | 68.8 | 76.7 | 83.2 |
| Age, years | 50.1 (10.2) | 55.8 (10.5) | 60.6 (9.8) | 64.0 (8.8) |
| Education level, % |  |  |  |  |
| Primary or below | 55.6 | 75.8 | 87.8 | 92.5 |
| Secondary or above | 44.4 | 24.2 | 12.2 | 7.5 |
| Household income, % |  |  |  |  |
| Low | 9.9 | 12.6 | 18.2 | 20.4 |
| Middle | 49.0 | 49.3 | 50.1 | 52.3 |
| High | 41.0 | 38.1 | 31.7 | 27.4 |
| Ever smoking, % | 4.8 | 5.8 | 5.2 | 5.8 |
| Regular alcohol use, % | 37.3 | 23.9 | 18.8 | 16.7 |
| Physical activity (MET hours/day) | 17.6 (11.2, 29.1) | 14.0 (9.1, 23.6) | 11.2 (8.4, 18.9) | 10.2 (8.4, 14.8) |
| Systolic blood pressure, mmHg | 129.0 (21.6) | 134.6 (23.5) | 139.4 (24.1) | 142.7 (24.7) |
| Body mass index, kg/m^2^ | 23.8 (3.4) | 23.3 (3.5) | 22.8 (3.5) | 22.4 (3.5) |
| History of hypertension, % | 10.0 | 14.2 | 18.3 | 17.2 |
| History of diabetes, % | 2.8 | 3.9 | 3.8 | 4.6 |
| Pregnancies, n | 3.2 (1.6) | 4.5 (5.9) | 5.9 (1.8) | 7.6 (2.2) |
| Pregnancy loss, % |  |  |  |  |
| History of miscarriage | 8.6 | 14.3 | 17.8 | 20.4 |
| History of induced abortion | 53.5 | 34.7 | 25.2 | 23.0 |
| Ever use of oral contraceptives, % | 10.1 | 6.6 | 6.0 | 4.3 |

**Table S4: Baseline characteristics of study participants by number of stillbirths**

Values are percentages for categorical variables, and means and standard deviations for continuous variables, expect for physical activity where median and 25^th^ and 75^th^ percentile are shown. MET, metabolic equivalent

|  | **Ischaemic stroke** | | | **Haemorrhagic stroke** | | |
| --- | --- | --- | --- | --- | --- | --- |
|  | **No. events** | **HR (95% CI)**  **Model I** | **HR (95% CI)**  **Model II** | **No. events** | **HR (95% CI)**  **Model I** | **HR (95% CI)**  **Model II** |
| **Pregnancies** |  |  |  |  |  |  |
| *Ever vs. never* |  | *0.92 (0.78; 1.10)* | *0.92 (0.78; 1.09)* |  | *1.01 (0.64; 1.58)* | *1.05 (0.67; 1.65)* |
| None | 133 | 1.12 (0.95; 1.33) | 1.11 (0.93; 1.31) | 19 | 1.04 (0.67; 1.64) | 0.99 (0.63; 1.55) |
| 1 | 563 | 1.00 (0.92; 1.09) | 1.00 (0.92; 1.09) | 75 | 1.00 (0.79; 1.27) | 1.00 (0.79; 1.27) |
| 2 | 1848 | 0.97 (0.93; 1.02) | 0.97 (0.92; 1.01) | 327 | 1.00 (0.89; 1.12) | 1.00 (0.89; 1.13) |
| 3 | 2651 | 1.02 (0.98; 1.06) | 1.01 (0.97; 1.05) | 486 | 1.05 (0.96; 1.15) | 1.05 (0.96; 1.15) |
| 4 | 2383 | 1.02 (0.98; 1.07) | 1.01 (0.97; 1.05) | 423 | 0.99 (0.90; 1.09) | 0.97 (0.89; 1.07) |
| ≥5 | 3852 | 1.10 (1.06; 1.14) | 1.07 (1.03; 1.11) | 840 | 1.10 (1.01; 1.20) | 1.06 (0.98; 1.16) |
| *Per additional^†^* |  | *1.02 (1.01; 1.04)* | *1.02 (1.01; 1.03)* |  | *1.02 (1.00; 1.05)* | *1.01 (0.99; 1.04)* |
| **Miscarriages** |  |  |  |  |  |  |
| *Ever vs. never^‡^* |  | *1.01 (0.95; 1.07)* | *1.03 (0.97; 1.09)* |  | *0.97 (0.86; 1.10)* | *0.98 (0.86; 1.11)* |
| None | 10108 | 1.00 (0.98; 1.02) | 1.00 (0.98; 1.02) | 1853 | 1.00 (0.95; 1.06) | 1.00 (0.95; 1.06) |
| 1 | 939 | 1.02 (0.96; 1.09) | 1.04 (0.97; 1.10) | 210 | 0.93 (0.81; 1.06) | 0.94 (0.82; 1.07) |
| 2 | 183 | 0.97 (0.84; 1.12) | 1.00 (0.86; 1.16) | 59 | 1.05 (0.81; 1.36) | 1.07 (0.83; 1.38) |
| ≥3 | 67 | 0.96 (0.75; 1.21) | 0.98 (0.77; 1.24) | 29 | 1.24 (0.86; 1.79) | 1.14 (0.78; 1.64) |
| *Per additional^†^* |  | *0.97 (0.89; 1.05)* | *0.98 (0.90; 1.07)* |  | *1.10 (0.97; 1.25)* | *1.07 (0.94; 1.22)* |
| **Induced abortions** | |  |  |  |  |  |
| *Ever vs. never^‡^* |  | *1.11 (1.06; 1.15)* | *1.06 (1.01; 1.10)* |  | *0.89 (0.80; 0.99)* | *0.94 (0.85; 1.04)* |
| None | 7334 | 1.00 (0.97; 1.03) | 1.00 (0.97; 1.03) | 1456 | 1.00 (0.93; 1.08) | 1.00 (0.93; 1.08) |
| 1 | 3714 | 1.08 (1.05; 1.11) | 1.04 (1.01; 1.08) | 381 | 0.87 (0.79; 0.96) | 0.91 (0.82; 1.00) |
| 2 | 2160 | 1.14 (1.09; 1.19) | 1.06 (1.01; 1.11) | 218 | 0.98 (0.85; 1.12) | 1.05 (0.91; 1.20) |
| ≥3 | 1066 | 1.15 (1.08; 1.22) | 1.09 (1.02; 1.17) | 96 | 0.80 (0.66; 0.99) | 0.86 (0.70; 1.06) |
| *Per additional^†^* |  | *1.03 (1.01; 1.06)* | *1.03 (1.00; 1.05)* |  | *0.97 (0.90; 1.05)* | *0.98 (0.91; 1.06)* |
| **Stillbirths** |  |  |  |  |  |  |
| *Ever vs. never^‡^* |  | *1.00 (0.94; 1.07)* | *1.04 (0.97; 1.12)* |  | *1.12 (0.97; 1.30)* | *1.08 (0.93; 1.25)* |
| None | 13078 | 1.00 (0.97; 1.04) | 1.00 (0.96; 1.04) | 1898 | 1.00 (0.92; 1.09) | 1.00 (0.92; 1.09) |
| 1 | 822 | 0.96 (0.90; 1.02) | 1.05 (0.98; 1.13) | 157 | 1.03 (0.88; 1.19) | 0.99 (0.86; 1.16) |
| 2 | 240 | 1.07 (0.94; 1.22) | 1.02 (0.87; 1.19) | 63 | 1.35 (1.05; 1.74) | 1.28 (0.99; 1.65) |
| ≥3 | 134 | 1.29 (1.08; 1.54) | 0.92 (0.71; 1.20) | 33 | 1.37 (0.96; 1.95) | 1.30 (0.92; 1.86) |
| *Per additional^†^* |  | *1.11 (1.05; 1.19)* | *0.98 (0.89; 1.08)* |  | *1.14 (1.03; 1.26)* | *1.13 (1.02; 1.26)* |

**Table S5: Adjusted hazard ratios (95% confidence intervals) for incident ischaemic and haemorrhagic stroke subtype associated with number of pregnancies, miscarriages, induced abortions, and stillbirths**

Model I: HRs are stratified by age at risk and study area. Model II: HRs are stratified by age and study area, and adjusted for level of attained education, household income, smoking status, alcohol use, systolic blood pressure, history of hypertension, physical activity, body mass index, and history of diabetes. Model II analyses for miscarriage, induced abortion, and stillbirth were additionally adjusted for number of live births, and where appropriate, number of miscarriages, induced abortions, and stillbirths. †Analyses are restricted to women with at least one pregnancy, miscarriage, induced abortion, or stillbirth, respectively. ‡Analyses are restricted to women with at least one pregnancy.

|  | **CHD** | | **Stroke** | | **Circulatory disease** | |
| --- | --- | --- | --- | --- | --- | --- |
|  | **No. events** | **HR (95% CI)** | **No. events** | **HR (95% CI)** | **No. events** | **HR (95% CI)** |
| **Pregnancies** |  |  |  |  |  |  |
| *Ever vs. never* |  | *0.93 (0.80; 1.08)* |  | *0.99 (0.86; 1.13)* |  | *0.98 (0.89; 1.08)* |
| None | 166 | 1.18 (1.01; 1.38) | 211 | 1.07 (0.93; 1.22) | 429 | 1.15 (1.04; 1.26) |
| 1 | 779 | 1.00 (0.93; 1.08) | 994 | 1.00 (0.94; 1.07) | 2333 | 1.00 (0.96; 1.04) |
| 2 | 2534 | 1.01 (0.97; 1.05) | 3501 | 0.99 (0.96; 1.03) | 8915 | 1.07 (1.04; 1.09) |
| 3 | 3306 | 1.04 (1.00; 1.07) | 4718 | 1.03 (1.00; 1.06) | 11193 | 1.11 (1.09; 1.13) |
| 4 | 3021 | 1.15 (1.11; 1.19) | 4064 | 1.04 (1.01; 1.07) | 8804 | 1.14 (1.11; 1.16) |
| ≥5 | 4634 | 1.21 (1.16; 1.25) | 6437 | 1.13 (1.09; 1.16) | 12294 | 1.21 (1.19; 1.24) |
| *Per additional^†^* |  | *1.04 (1.03; 1.06)* |  | *1.03 (1.02; 1.04)* |  | *1.03 (1.03; 1.04)* |
| **Miscarriages** |  |  |  |  |  |  |
| *Ever vs. never^‡^* |  | *1.07 (1.02; 1.13)* |  | *1.04 (1.00; 1.09)* |  | *1.03 (1.00; 1.06)* |
| None | 12629 | 1.00 (0.98; 1.02) | 17497 | 1.00 (0.98; 1.02) | 39089 | 1.00 (0.99; 1.01) |
| 1 | 1220 | 1.04 (0.98; 1.10) | 1695 | 1.03 (0.98; 1.08) | 3390 | 1.01 (0.98; 1.04) |
| 2 | 304 | 1.17 (1.04; 1.31) | 374 | 1.07 (0.96; 1.18) | 779 | 1.10 (1.03; 1.18) |
| ≥3 | 121 | 1.24 (1.03; 1.48) | 148 | 1.14 (0.97; 1.34) | 281 | 1.10 (0.98; 1.24) |
| *Per additional^†^* |  | *1.06 (1.00; 1.13)* |  | *1.03 (0.98; 1.09)* |  | *1.04 (1.00; 1.08)* |
| **Induced abortions** | |  |  |  |  |  |
| *Ever vs. never^‡^* |  | *1.11 (1.06; 1.15)* |  | *1.04 (1.01; 1.07)* |  | *1.04 (1.02; 1.06)* |
| None | 7334 | 1.00 (0.97; 1.03) | 10071 | 1.00 (0.98; 1.02) | 22775 | 1.00 (0.98; 1.02) |
| 1 | 3714 | 1.08 (1.05; 1.11) | 5217 | 1.03 (1.00; 1.06) | 11834 | 1.03 (1.01; 1.05) |
| 2 | 2160 | 1.14 (1.09; 1.19) | 2904 | 1.04 (1.00; 1.08) | 6032 | 1.04 (1.01; 1.07) |
| ≥3 | 1066 | 1.15 (1.08; 1.22) | 1522 | 1.09 (1.04; 1.15) | 2898 | 1.08 (1.04; 1.12) |
| *Per additional^†^* |  | *1.03 (1.01; 1.06)* |  | *1.03 (1.01; 1.05)* |  | *1.02 (1.01; 1.04)* |
| **Stillbirths** |  |  |  |  |  |  |
| *Ever vs. never^‡^* |  | *1.00 (0.94; 1.07)* |  | *1.06 (1.01; 1.12)* |  | *1.07 (1.03; 1.11)* |
| None | 13078 | 1.00 (0.97; 1.04) | 17846 | 1.00 (0.97; 1.03) | 40052 | 1.00 (0.98; 1.02) |
| 1 | 822 | 0.96 (0.90; 1.02) | 1355 | 1.05 (1.00; 1.11) | 2538 | 1.05 (1.01; 1.09) |
| 2 | 240 | 1.07 (0.94; 1.22) | 368 | 1.12 (1.01; 1.25) | 662 | 1.12 (1.04; 1.21) |
| ≥3 | 134 | 1.29 (1.08; 1.54) | 145 | 0.99 (0.84; 1.17) | 287 | 1.12 (0.99; 1.26) |
| *Per additional^†^* |  | *1.11 (1.05; 1.19)* |  | *1.01 (0.95; 1.07)* |  | *1.03 (0.99; 1.08)* |

**Table S6: Adjusted hazard ratios (95% confidence intervals) for incident coronary heart disease, stroke, and circulatory disease associated with number of pregnancies, miscarriages, induced abortions, and stillbirths**

HRs are stratified by age at risk and study area. †Analyses are restricted to women with at least one pregnancy, miscarriage, induced abortion, or stillbirth, respectively. ‡Analyses are restricted to women with at least one pregnancy.

**Figure S1: Adjusted hazard ratios (95% confidence intervals) for incident circulatory disease associated with number of pregnancies, miscarriages, induced abortions, and stillbirths**


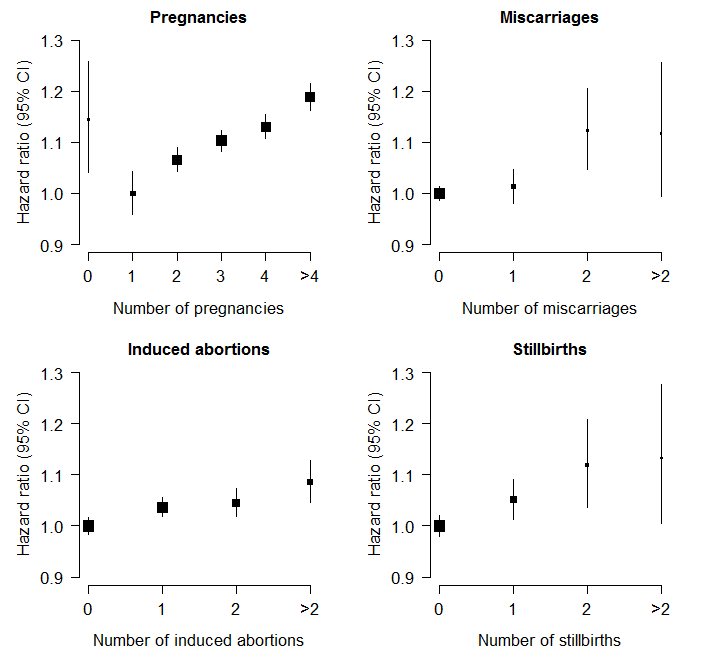


**Figure S2: Adjusted hazard ratios (95% confidence intervals) for incident coronary heart disease and stroke associated with number of pregnancies, miscarriages, induced abortions, and stillbirths**

**Figure S3: Adjusted hazard ratios for incident CHD and stroke per additional pregnancy by baseline characteristics**

**Figure S4: Adjusted hazard ratios for incident CHD and stroke per additional miscarriage by baseline characteristics**

**Figure S5: Adjusted hazard ratios for incident CHD and stroke per additional induced abortion by baseline characteristics**

**Figure S6: Adjusted hazard ratios for incident CHD and stroke per additional stillbirth by baseline characteristics**
